# Efficacy and safety of toludesvenlafaxine hydrochloride sustained-release tablets in depression with ineffective or partially effective initial antidepressant treatment: a single-arm, multicenter clinical study

**DOI:** 10.3389/fpsyt.2025.1740789

**Published:** 2026-02-05

**Authors:** Jingming Yang, Yanyan Jia, Ke Chen, Weiye Liang, Jing Long, Meijuan Li, Qianqian Wei, Jian Wang, Baoping Yan, Keqing Li, Jie Li, Fude Yang

**Affiliations:** 1 Department of Psychiatry, Beijing Huilongguan Hospital, Peking University Huilongguan Clinical Medical College, Beijing, China; 2 Department of Psychiatry, Beijing Changping Mental Health Hospital, Beijing, China; 3 Department of Psychology, Tianjin Anding Hospital, Tianjin, China; 4 Department of Pharmacology, Hebei Provincial Mental Health Center, The Sixth Clinical Medical College of Hebei University, Baoding, Hebei, China

**Keywords:** clinical efficacy, initial antidepressant, switching strategies, toludesvenlafaxine, treatment-resistant depressive disorder

## Abstract

**Purpose:**

Depression is one of the leading causes of avoidable suffering worldwide, and over 50% of patients do not respond to their first antidepressant treatment, which underscores the need for more effective alternatives. This clinical predicament urgently requires an effective solution. The core objective of this study is to clarify the clinical efficacy and application value of the triple reuptake inhibitor toludesvenlafaxine in patients with poor response to the first antidepressant therapy, providing a new basis for treatment options for this refractory group.

**Methods:**

This multicenter study included 61 patients meeting *Diagnostic and Statistical Manual of Mental Disorders, Fifth Edition* (*DSM-V*) criteria for depression. All patients still had a Montgomery Depression Rating Scale (MADRS) score of ≥24 after 4 weeks of treatment with an adequate single initial antidepressant, and were clearly classified as an ineffective or partially effective initial antidepressant treatment. Patients were switched to toludesvenlafaxine for an 8-week treatment period. The primary outcome measure was the change in MADRS score from baseline to 8 weeks. The secondary outcome measures included the changes in the scores of the Hamilton Anxiety Scale (HAMA), the Dimensional Anhedonia Rating Scale (DARS), the Quality of Life Enjoyment and Satisfaction Questionnaire, Short Form (Q-LES-Q-SF), and the Clinical Global Impression of Severity Scale (CGI-S) from baseline to 8 weeks.

**Results:**

For such patients that did not respond to the first treatment, significant and rapid efficacy was demonstrated after switching to toludesvenlafaxine. At 8 weeks of treatment, the average MADRS score of the patients decreased by 15.5 points compared with the baseline (95% CI, −17.7 to −13.3; *p* < 0.0001; Cohen’s *d* = 2.35). Forty-three percent of them met the clinical remission, and 67% achieved a clinical response. More clinically significant is that the therapeutic effect emerged at an early stage—2 weeks (95% CI, −9.4 to −6.4; *p* < 0.0001; Cohen’s *d* = 1.52) and 4 weeks (95% CI, −13.3 to −9.8; *p* < 0.0001; Cohen’s *d* = 2.11). After 2 weeks of treatment, there were statistically significant differences in the HAMA, Q-LES-Q-SF, and CGI-S scores at each time point compared with the baseline. The improvement in the DARS score was statistically significant from 4 weeks. In terms of safety, the most common adverse reactions were palpitations, constipation, nausea, vomiting, hypoesthesia, and dizziness, which are mostly mild to moderate and controllable. In particular, the drug significantly improved sexual dysfunction (95% CI, −4.3 to −0.7; *p* = 0.0071), which is crucial for improving treatment compliance.

**Conclusion:**

This study confirmed that toludesvenlafaxine not only has significant clinical efficacy (including early onset, high remission, and response) for patients with depression who did not respond to the first antidepressant treatment, but also has good safety and can improve sexual dysfunction that affects compliance. This result highlights the significant position of toludesvenlafaxine in addressing the key clinical challenge of first treatment failure, providing a highly valuable new option for the subsequent treatment of such patients.

## 1 Introduction

The World Health Organization (WHO) predicts that depressive disorder will become the number one cause of disease burden in the world by 2030 (1). Currently, approximately 5.0% of adults worldwide suffer from depression, and it has become one of the main causes of disability. It is worth noting that depression is the leading cause of avoidable suffering worldwide, which means that early and effective intervention can significantly reduce its harm to individuals, families, and society (2). The rational drug change after the failure of initial antidepressant treatment is precisely the key link to achieve this goal, but it is also a prominent problem in current clinical practice.

Failure of initial antidepressant treatment represents a pressing clinical dilemma in managing treatment-resistant depression (TRD), which is a complex condition with multifactorial origins, including neurobiological, genetic, and environmental factors (3). Beyond neurotransmitter imbalances, emerging research points to glymphatic system dysfunction as a potential contributor to depression and treatment resistance (4). By regulating metabolic waste clearance and brain homeostasis, glymphatic impairment may hinder neuroplasticity and toxin elimination, further complicating TRD pathogenesis and underscoring the need for multifaceted therapies. However, pharmacotherapy remains the core option of depression treatment, and combination therapy is only adopted when full-dose, full-course single-drug treatment and drug switching have failed (5). Studies show that only 11%–30% of patients can achieve recovery after 8 to 12 months of single antidepressant treatment (6, 7), and fewer than 50% experience symptom relief with the first therapy (8, 9). Thus, switching is the primary strategy for patients with poor initial response. Although minimal improvement by 2 weeks may predict ultimate failure (10), clinical guidelines and comprehensive consensus recommend waiting at least 4 weeks to evaluate response (11–13). Switching too early may miss the potential efficacy of the initial drug, while delaying may prolong the ineffective treatment and miss the best intervention window. The essence of this contradiction is the reality of the lack of effective alternative drugs.

At present, the recommended alternative drugs are still traditional antidepressants such as selective serotonin reuptake inhibitors (SSRIs) and serotonin–norepinephrine reuptake inhibitors (SNRIs) (2, 14–16). However, these agents have limited dopaminergic activity, which may contribute to persistent symptoms. As a key regulator of pleasure, motivation, and cognition, dopamine insufficiency perpetuates residual symptoms (anhedonia, reduced motivation, and cognitive deficits) that hinder functional recovery and erode treatment adherence (17). While these conventional drugs remain clinically used, their limitations reflect an unmet need for alternatives targeting broader neurochemical pathways (18). Novel therapeutic approaches in TRD (intranasal esketamine) have shown promise in the real-world and clinical settings, demonstrating safety and effectiveness for some patients (19–21). However, access, tolerability, and individual variability in response may highlight the need for additional treatment options. Toludesvenlafaxine, a new triple reuptake inhibitor (TRI), differs from traditional agents by simultaneously inhibiting the reuptake of 5-hydroxytryptamine (5-HT), norepinephrine (NE), and dopamine (DA) (22, 23). This mechanism is designed to address the broader neurochemical imbalances implicated in depression, including DA-related deficits that may contribute to residual symptoms (22, 24). While the systematic review and meta-analysis data support its efficacy compared to other antidepressants (24), direct evidence for its performance in patients with initial treatment failure (an important subset of TRD) is lacking.

Despite toludesvenlafaxine’s mechanistic promise and the unmet needs in TRD management, no studies have specifically examined its clinical outcomes in patients who failed the initial antidepressant therapy. Clinicians thus lack evidence-based guidance for using toludesvenlafaxine as an alternative, while patients face limited options beyond traditional agents with proven limitations. The present study addresses the evidence gap by systematically evaluating the efficacy and safety of toludesvenlafaxine in patients with ineffective or partially effective initial antidepressant treatment. By focusing on this specific TRD cohort, we aim to provide an evidence-based alternative for clinical practice, reduce trial and error in treatment adjustment, and offer insights into personalized strategies for a patient group with unmet needs.

## 2 Methods

### 2.1 Study design and subjects

The study was designed as an open-label, single-arm, multicenter study to evaluate the efficacy and safety of toludesvenlafaxine in depressed patients with ineffective or partially effective initial antidepressant treatment. A CONSORT-style rationale for the absence of a control group is as follows. Given the ethical considerations of exposing this refractory population to a placebo that would prolong ineffective treatment and potentially exacerbate symptoms, including suicide risk, an open-label single-arm design was deemed appropriate. Secondly, this study focused on generating preliminary efficacy and safety data for toludesvenlafaxine in this specific subgroup, which has been understudied. Furthermore, the use of well-validated assessment scales allowed for robust within-group comparisons, and the results were contextualized against published data to ensure interpretability.

The sample size calculation was based on the primary endpoint of change in the Montgomery–Asberg Depression Rating Scale (MADRS) total score from baseline to week 8. The mean paired difference is 10 and the standard deviation (SD) of paired differences is 13 (power = 0.9, αlevel = 0.0001), according to a previous study related to new antidepressants (25). The results showed that the sample sizes should be 52 considering the 20% dropout rate.

We recruited patients aged 22 to 65 years from February to July 2024 at three hospitals in China, meeting the criteria for depression disorder according to the *Diagnostic and Statistical Manual of Mental Disorders, Fifth Edition* (*DSM-V*) and 32-item Hypomania Check list (HCL-32, score < 14). These patients not only had been treated with one enough antidepressant for at least 4 weeks, but also had at least a score of 24 in the MADRS. All patients provided informed consent. Exclusion criteria included the following: (i) with psychotic symptoms; (ii) serious self-injury, apparent suicide attempt or behavior, and MADRS item 10 (suicidal thoughts) score ≥4; (iii) have received ECT or rTMS in the past 3 months; (iv) with antipsychotics and mood stabilizers; and (v) alcohol/substance abuse, active medical or neurological problems, and other psychiatric diseases. Patients could be free to withdraw from the trial for any reason throughout the study, and investigators also had the right to remove patients from the study for any safety-related reason. This study was approved by the ethics committee of three centers. In this single-arm study, partial blinding method was implemented. The blinding of treating investigators is infeasible because of the same intervention. Outcome assessments were conducted by independent blind assessors who had no involvement in any aspects of patient treatment or management.

### 2.2 Study endpoints

The strategy of cross-medication was adopted in this study (26). The original drug was discontinued within 2 weeks and replaced with toludesvenlafaxine. The recommended initial dose is 40 mg, which can be increased to 80 mg per day within 1 week according to the patient’s tolerance, and the maximum therapeutic dose should not exceed 160 mg per day (24). The recommended therapeutic dose is 80 to 160 mg daily, which can be lowered during the increase if the patient is intolerant. Researchers selected the dosage according to the actual condition of the patients. The drug was administered for 8 weeks. Toludesvenlafaxine should be taken at a relatively fixed time each day and can be taken orally on an empty stomach or after meals, once a day. To assess the efficacy accurately, participants were not allowed to use benzodiazepines and only used sleep aids for no more than 2 consecutive weeks.

Four evaluation nodes were set up in this study, which were at the time of baseline, week 2, week 4, and week 8, respectively. The primary endpoint was the change from baseline to week 8 in the MADRS total score, which reflects changes in depressive symptoms and assesses the effect of antidepressant therapy (27). The MARDS has 10 items, with overall scores ranging from 0 to 60. The change in the MADRS total score from baseline to weeks 2 and 4 was the key secondary endpoint.

Other secondary endpoints included the Hamilton Anxiety Scale [HAMA; scores range from 0 (none) to 4 (extremely severe)] (28); the Dimensional Anhedonia Rating Scale [DARS; including four dimensions: A, hobbies; B, food/drink; C, social activities; and D, sensory experiences; scores range from 0 (none) to 4 (always)] (29); the Quality of Life Enjoyment and Satisfaction Questionnaire, Short Form (Q-LES-Q-SF; scores range from 1 to 5, with higher scores representing higher satisfaction) (30); and the Clinical Global Impression of Severity Scale [CGI-S; scores range from 1 (normal state) to 7 (among the most extremely ill)] (31).

Adverse events were recorded in vital signs, clinical tests, physical examination, and electrocardiogram, to evaluate the safety of experimental drugs. Adverse events during the trial were recorded from the first oral toludesvenlafaxine to the last visit. Adverse effects on sexual function were assessed using the Arizona Sexual Experience Scale (ASEX), ranging from 1 to 6, with higher scores representing more severe sexual dysfunction (32).

### 2.3 Statistical analysis

A full analysis set (FAS) is a collection of eligible and shedding cases, but excludes excluded cases. As the primary efficacy measure, missing data for MADRS scores in the FAS were handled using the Last Observation Carried Forward (LOCF) method for intention-to-treat (ITT) analysis. As the secondary efficacy scales (HAMA, DARS, Q-LES-Q-SF, and CGI-S), analyses were restricted to patients with complete data at each assessment time point in the FAS. The safety analysis set (SAS) should include safety measures for all treated patients, despite having received only one dose of the study medication. Safety loss value shall not be carried forward. The incidence of adverse reactions was analyzed on the basis of the safety set.

All statistical analyses will be programmed using SAS Version 9.4 Statistical analysis software. Paired *t*-test was used for within-group comparison. A two-sided α level of 0.05 will be considered a hypothesis test, and 95% confidence is used for the confidence interval.

## 3 Results

### 3.1 Demographic characteristics

**Figure 1:**
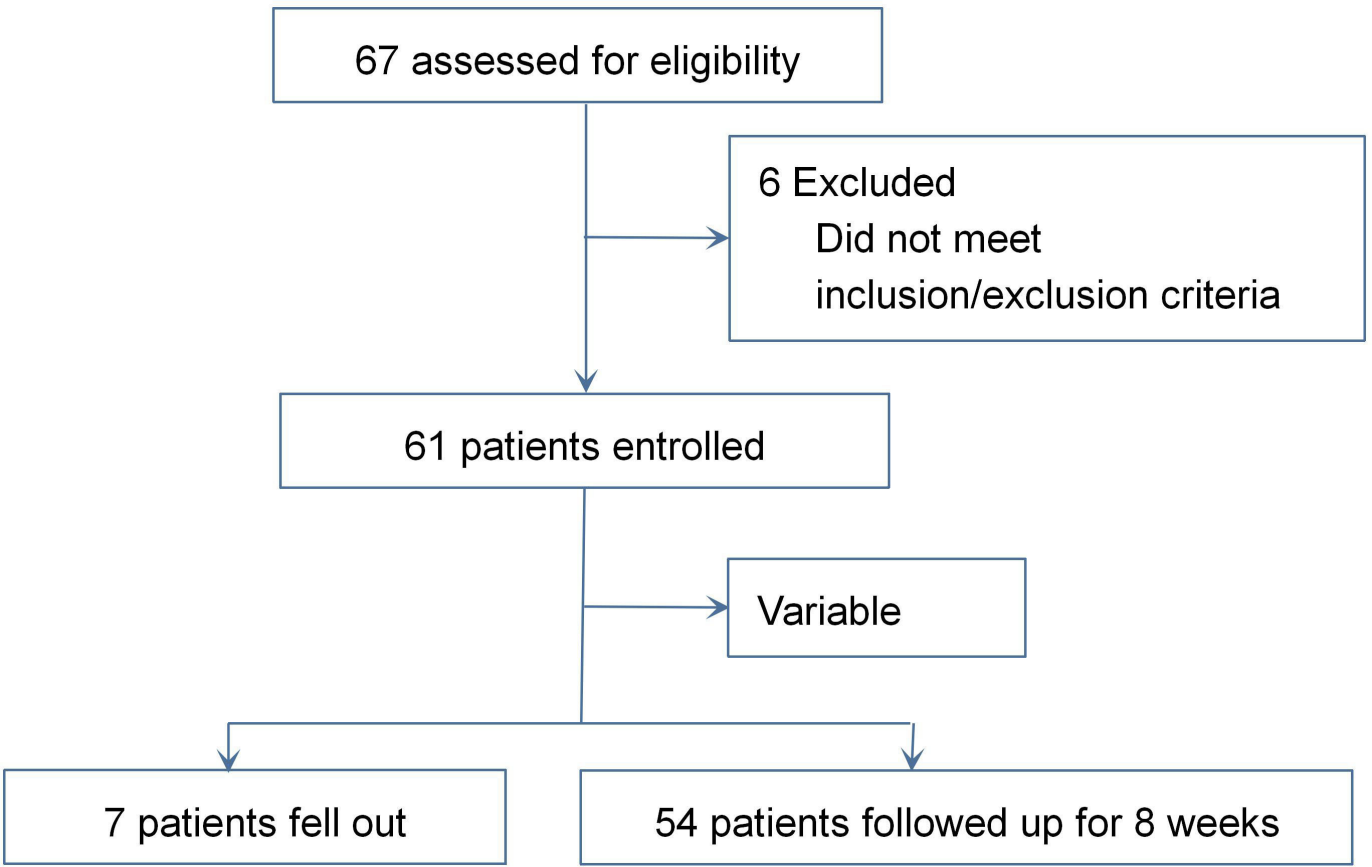
The flow of sample size.

After screening, 61 patients who met the above criterion received the experimental drug. Seven cases dropped out due to various reasons, and 54 cases completed full follow-up (**Figure 1**). Of the seven patients who dropped out, two were considered unsuitable for the study drug because of an increased risk of suicide. The other five cases were discontinued due to protocol deviations, primarily including non-adherent medication administration and missed scheduled study visits beyond the predefined time windows. The mean age of all enrolled patients (female patients, 44) was 36.9 years, and the duration of the disease was 4.04 years. All blood pressure and heart rate were normal. Baseline demographic and clinical features of completers (*n* = 54) and non-completers (*n* = 7) were compared. Completers: age, 37.2 ± 9.8 years; 74.1% female; disease duration, 4.1 ± 3.2 years; baseline scores: MADRS, 32.6 ± 4.5; HAMA, 28.3 ± 6.2; DARS, 42.5 ± 12.3; Q-LES-Q-SF, 28.6 ± 5.4; CGI-S, 4.3 ± 0.6; and ASEX, 18.2 ± 4.6. Non-completers: age, 35.1 ± 8.5 years; 57.1% female; disease duration, 3.8 ± 2.9 years; baseline scores: MADRS, 33.8 ± 3.9; HAMA, 29.5 ± 5.8; DARS, 45.2 ± 10.7; Q-LES-Q-SF, 27.3 ± 6.1; CGI-S, 4.5 ± 0.5; and ASEX, 19.5 ± 3.8. No statistically significant differences were noted between groups across all assessed characteristics (*p* > 0.05).

### 3.2 Efficacy

**Figure 2:**
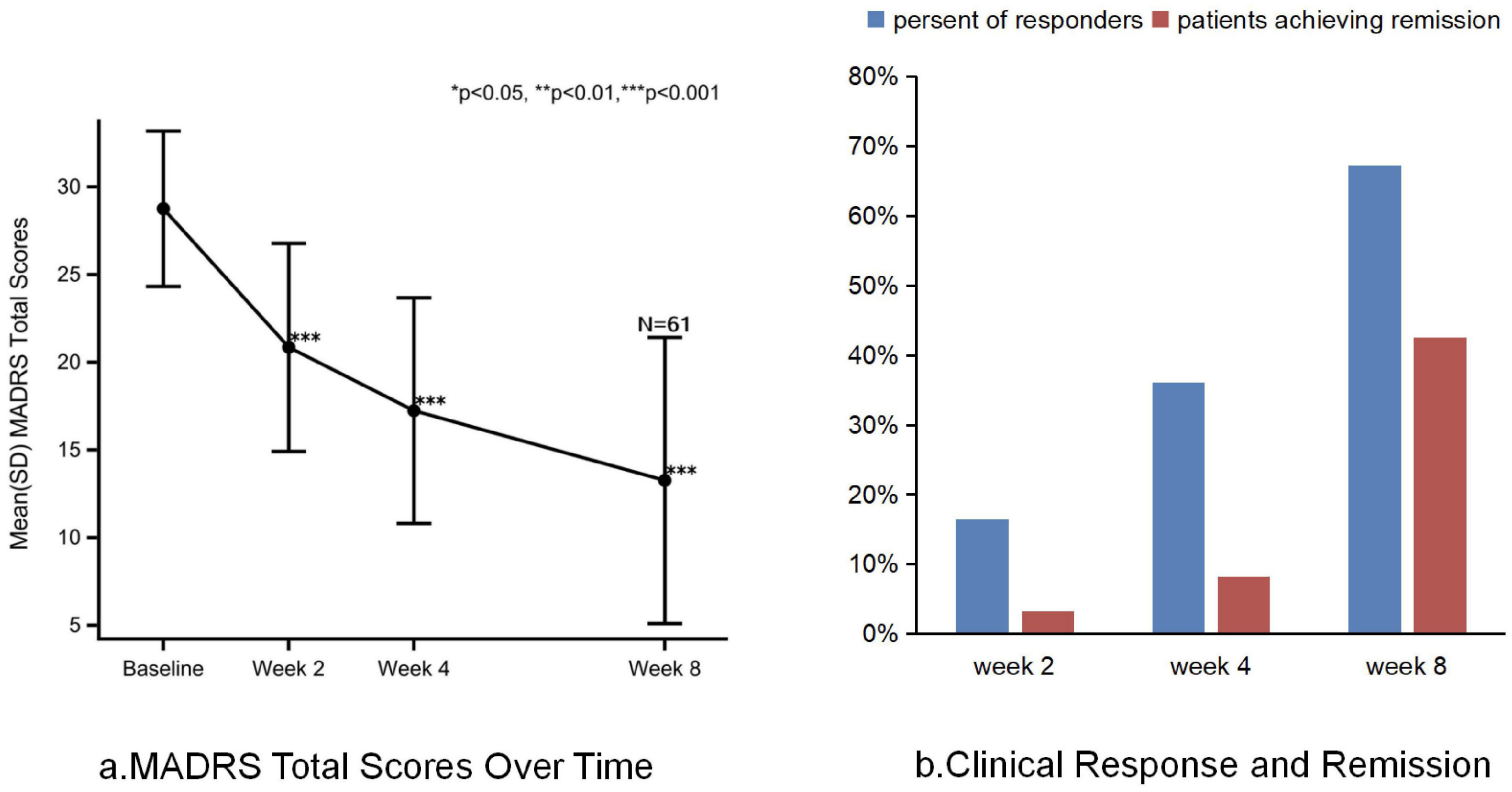
MADRS total score, clinical response and remission after treatment with toludesvenlafaxine. **(a)** MADRS total score over time. **(b)** Clinical response and remission.

Compared to baseline, switching to toludesvenlafaxine significantly reduced the total MADRS score at all assessment time points (*p* < 0.0001). From baseline to week 8, the mean change in total MADRS score after switching to toludesvenlafaxine was −15.5 points [95% confidence interval (CI), −17.7 to −13.3; Cohen’s *d* = 2.35]. Sensitivity analysis results for changes in MADRS scores from baseline to week 8 also showed statistical significance (mean ± SD −17.6 ± 6.3; 95% CI, −19.4 to −15.9; Cohen’s *d* = 3.22). At the first time point, week 2 of the trial, the mean change in the total MADRS score from baseline after switching to toludesvenlafaxine was −7.90 points (95% CI, −9.4 to −6.4; Cohen’s *d* = 1.52). At week 4, the mean change in total MADRS score from baseline for toludesvenlafaxine was −11.5 points (95% CI, −13.3 to −9.8; Cohen’s *d* = 2.11) (**Figure 2a**). Clinical response was defined as a ≥50% reduction in MADRS total score. Remission was defined as a MADRS total score ≤10. At week 8, 67% of patients achieved clinical response (95% CI, 54.0 to 78.7), and 43% of patients achieved remission (95% CI, 30.0 to 55.9) (**Figure 2b**). Referring to the corresponding data of traditional antidepressant drug switching, the number needed to treat (NNT) for clinical remission was 5.55 and the NNT for clinical response was 3.7 (8, 12, 18, 33).

The total score of CGI-S in patients treated with toludesvenlafaxine decreased significantly from baseline at all time points assessed. Moreover, the total score showed a downward trend from week 2 to week 8 after medication with a statistically significant difference (**Table 1**). The mean change from baseline to week 8 was −1.9 (*p* < 0.0001). The disease severity of all patients was moderate or above at baseline, and the proportion of patients with disease severity below moderate was 70.9% at week 8. At all time points, the decrease on the HAMA’s total score was statistically significant from baseline. The change in the total score of DARS with toludesvenlafaxine was observed from week 4. From week 2 after treatment, the total DARS score tended to increase from baseline, but the statistical difference was not significant (*p* = 0.0509). The Q-LES-Q-SF score of patients treated with toludesvenlafaxine was significantly higher than baseline starting from week 2, and further increased to week 8, with statistically significant differences from baseline (*p* < 0.0001).

**Table 1:** Secondary outcomes after treatment with toludesvenlafaxine.

Score change from baseline	Week 2	Week 4	Week 8
HAMA	−5.6 ± 6.4^**^	−8.9 ± 6.5^**^	−12.7 ± 7.6^**^
DARS	2.7 ± 10.2	6.7 ± 13.3^*^	9.1 ± 14.8^**^
Q-LES-Q-SF	3.7 ± 6.4^**^	7.1 ± 8.3^**^	8.9 ± 9.2^**^
CGI-S	−0.7 ± 0.7^**^	−1.2 ± 0.8^**^	−1.9 ± 1.1^**^

*p < 0.001; **p < 0.0001.

### 3.3 Safety

The percentage of adverse events during treatment with toludesvenlafaxine was 16.4%, of which the rate of drug-related adverse events was 11.5%. The common adverse reactions related to the study drugs were as follows: palpitations, constipation, nausea, vomiting, hypoesthesia, and dizziness. There was only one severe adverse event (aggravated depression) in the entire trial that the investigator determined was not related to the study drug and chose to discontinue the study drug based on the patient’s condition. During the study period, the incidence of adverse events associated with study drug “elevated blood triglycerides” in laboratory results was 1.6% and the severity was mild (**Table 2**). After 8 weeks, no statistical differences were shown in terms of weight (95% CI, −0.82 to 0.55; *p* = 0.6988) and blood pressure (systolic 95% CI, −6.3 to 0.3; *p* = 0.0762; diastolic 95% CI, −1.4 to 3.6; *p* = 0.3920) compared to baseline. At all assessment points, ASEX scores declined from baseline. There was a statistically significant decrease from baseline to week 2 in the total ASEX score (95% CI, −3.6 to −0.7; *p* = 0.0045) and a further decrease from baseline after week 8 (95% CI, −4.3 to −0.7; *p* = 0.0071) (**Table 3**).

**Table 2:** Summary of adverse events of toludesvenlafaxine for depressive disorder.

Type of adverse event	Toludesvenlafaxine (*N* = 61)
Any adverse event, *n* (%)	10 (16.1%)
Severe adverse event, *n* (%)	1 (3.3%)
Drug-related adverse event, *n* (%)	7 (11.5%)
Adverse events leading to discontinuation of study drug, *n* (%)	1 (3.3%)
Adverse events occurring in ≥3% of patients	
Gastrointestinal (constipation, nausea, and vomiting)	3 (4.9%)
Cardiovascular (palpitation)	2 (3.3%)
Nervous system (hypoesthesia and dizziness)	2 (3.3%)

**Table 3:** Some adverse events of toludesvenlafaxine for depressive disorder.

Type of adverse event	Toludesvenlafaxine (*N* = 61)
Change from baseline in ASEX at week 8, mean ± SD	−2.5 ± 6.3
Change from baseline in heart rate at week 8, mean ± SD, beats/min	0.4 ± 12.2
Change from baseline in body weight at week 8, mean ± SD, kg	−0.13 ± 2.45
Change from baseline in systolic at week 8, mean ± SD, mmHg	−3.0 ± 11.8
Change from baseline in diastolic at week 8, mean ± SD, mmHg	1.1 ± 8.9

## 4 Discussion

In this multicenter study, the antidepressant efficacy of switching to the TRI toludesvenlafaxine for patients who failed the initial treatment was confirmed to be statistically significant at both the primary and secondary study endpoints. The therapeutic value of toludesvenlafaxine in this specific population has been proven to be prominent. Even if the first antidepressant treatment was ineffective, MADRS scores declined progressively across all assessment points, indicating continuous symptom improvement. This indicates not only that toludesvenlafaxine brings rapid improvement to patients who do not respond to the first treatment, but also that its effect in improving symptoms can be steadily enhanced over time. Although some antidepressants have also been shown to have the characteristic of rapid onset, very few drugs can clearly reproduce this advantage in the first-treatment failure population (34). The results warrant contextualization within the broader landscape of treatments for difficult-to-treat depression.

The key unmet need in the management of TRD is early symptom relief to improve compliance and prevent adverse consequences. The efficacy of toludesvenlafaxine in this specific population has a certain degree of alignment with this demand. This echoes recent real-world evidence on intranasal esketamine. The Italian multicenter study, including the Realesk cohort, machine learning analysis of response predictors, and natural comparison between unipolar and bipolar TRD, consistently emphasized the clinical value of early improvement and treatment for residual symptoms such as lack of pleasure, anxiety, and reduced quality of life (19–21). Like esketamine, toludesvenlafaxine addresses these critical symptomatic domains. Studies have found that patients with depression with obvious anxiety before treatment have poorer curative effect (35). In this trial, it was found that the HAMA scores were statistically different at week 2 and at all subsequent time points compared with the baseline after switching to toludesvenlafaxine. The DAR scores showed that a statistically significant benefit was observed in DARS scores from week 4. This indicates that the overall function of the patient can be improved, because the improvement of anhedonia is also a strong predictor of improved psycho-social function (36). Additionally, quality of life (Q-LES-Q-SF) was also enhanced persistently and the severity of the disease (CGI-S) has gradually decreased from week 2 onward. The improvement of these multi-dimensional symptoms suggested that toludesvenlafaxine is an alternative drug in novel intervention measures given the failure of first-line treatment. Moreover, compared with the intranasal administration of esketamine, the oral formulation of toludesvenlafaxine may improve accessibility.

Among the specific patients, 67% achieved a clinical response and 43% achieved clinical remission after 8 weeks with toludesvenlafaxine. Although these data are slightly lower than those of the placebo-controlled Phase III clinical study, for patients who failed their first antidepressant treatment, the result is significantly better than that of other antidepressants in the same scenario (response rate, 40% to 50%; remission rate, <30%) (25, 33). The Sequential Treatment Study of Depression (STAR*D) reported that switching from an initial SSRI to another antidepressant yielded a response rate of 28%–33% and a remission rate of 15%–20% in Level 2 (first switch) (37, 38). Similarly, switching to bupropion (a norepinephrine–dopamine reuptake inhibitor) in STAR*D Level 2 resulted in a remission rate of 21%, with a response rate of 32% (37). As a new type of antidepressant drug with a mechanism of action different from toludesvenlafaxine, dextromethorphan-bupropion takes the antagonism of N-methyl-D-aspartate (NMDA) receptors and the activation of σ1 receptors as its core functions. Its clinical efficacy data showed that remission was achieved by 39.5% of patients and clinical response was attained by 54.0% (39). These comparisons highlight that toludesvenlafaxine’s efficacy outcomes are not only robust for an uncontrolled study but also competitive with established and emerging antidepressants. A targeted literature review based on the STAR*D study showed that increasing remission early in the disease trajectory may improve long-term outcomes (40). Our results initially confirmed that toludesvenlafaxine may have such an advantage.

As a TRI, toludesvenlafaxine simultaneously inhibits the reuptake of 5-HT, NE, and DA (23, 41), addressing a key limitation of traditional single or dual-channel antidepressants. Its targeted regulation of the DA system directly addresses dopamine hypofunction, a core contributor to treatment resistance and residual symptoms like anhedonia and reduced motivation (17). This mechanism likely underlies the observed improvement in DARS scores in this study, as DA modulation is critical for restoring reward processing and pleasure perception (42). Additionally, DA reuptake inhibition may counteract 5-HT-related side effects such as sexual dysfunction (43, 44), which is supported by our finding of significant reduction in the ASEX score. Beyond classical monoamine paradigms, emerging frameworks highlight neuroinflammatory, neurovascular, and glymphatic mechanisms in treatment resistance (4). While toludesvenlafaxine’s direct effects on these pathways remain unstudied, its multimodal monoamine regulation may indirectly influence these processes.

In this trial, toludesvenlafaxine was shown to be safe and well tolerated. Only 11.5% of patients experienced drug-related adverse events, with no drug-related severe adverse events or discontinuation. Common adverse reactions (palpitations, constipation, and nausea) were mild to moderate, consistent with SSRIs/SNRIs (37, 38). Notably, toludesvenlafaxine had no significant effects on body weight, blood pressure, or heart rate, and improved sexual function. It is a critical advantage given that sexual dysfunction is a major driver of non-adherence to traditional antidepressants (22). This safety profile compares favorably to esketamine, which is associated with transient dissociative symptoms, hypertension, and urinary tract symptoms in real-world use (19, 21), and to bupropion, which carries a risk of seizures at higher doses (12). The balanced efficacy–safety profile of toludesvenlafaxine enhances its clinical utility for long-term management of major depressive disorder.

## 5 Limitations

The study’s primary limitation is its single-arm, open-label design, which compromises causal inference. Without a control group (placebo or active control), improvements cannot be definitively attributed to toludesvenlafaxine. They may reflect regression to the mean, spontaneous improvement (natural history of depression), expectancy effects, or non-specific therapeutic factors (45). This is particularly relevant in antidepressant switching contexts, where early symptomatic improvement can occur due to discontinuation effects of the prior drug and engagement with a new treatment (10, 45). Additionally, reliance on the LOCF for missing MADRS data may introduce bias, especially given the small sample size and fluctuating nature of depressive symptoms. Other limitations include the narrow age range, precluding generalizability to adolescents or older adults with initial treatment failure. The study also did not analyze the type of initial antidepressant, which may influence switching outcomes (12, 33). Mechanistic claims regarding its dopamine function superiority over traditional agents, while supported by preclinical data (23, 41), lack direct comparative clinical evidence. Therefore, we will carry out targeted future research to enhance generalization such as recruiting diverse populations, conducting practical experiments, and expanding follow-ups.

## 6 Conclusion

Toludesvenlafaxine’s efficacy in our multicenter sample of initial treatment failure patients suggests potential generalizability to routine clinical settings, particularly for adults with moderate-to-severe depression and comorbidity anxiety or anhedonia. However, generalizability is limited by the exclusion of patients with psychotic symptoms, recent ECT/rTMS, or substance abuse, because these populations may have different treatment responses. The good safety and oral administration route of toludesvenlafaxine may make it a practical option for outpatient visits, complementing injection or nasogastric esketamine-based TRD strategies. For clinicians, toludesvenlafaxine can offer a targeted alternative to “trial-and-error” switching, reducing the time and burden of repeated treatment adjustments (46). In conclusion, toludesvenlafaxine has demonstrated the relatively comprehensive efficacy and good tolerability in patients with inadequate response to initial antidepressant treatment. Despite limitations inherent to the single-arm design, the study provided some valuable lines of evidence for toludesvenlafaxine as a promising option for difficult-to-treat depression, supporting the need for future randomized controlled trials to confirm its superiority over active controls and further explore its mechanistic underpinnings in the context of contemporary depression pathophysiology.

## Data Availability

The raw data supporting the conclusions of this article will be made available by the authors, without undue reservation.

## References

[1] Malhi GS Mann JJ . Depression. Lancet. (2018) 392:2299–312. doi: 10.1016/S0140-6736(18)31948-2, PMID: 30396512

[2] Herrman H Pateln V Kieling C Berk M Buchweitz C Cuijpers P . Time for united action on depression:a Lancet-World Psychiatric Association Commission. Lancet. (2022) 399:957–1022. doi: 10.1016/S0140-6736(21)02141-3, PMID: 35180424

[3] Di Vincenzo M Martiadis V Rocca DB Arsenio E D`Arpa A Volpicelli A . Facts and myths about use of esketamine for treatment-resistant depression: a narrative clinical review. Front Psychiatry. (2024) 15:1394787. doi: 10.3389/fpsyt.2024.1394787, PMID: 38812489 PMC11133709

[4] Barlattani T Cavatassi A Bologna A Socci V Trebbi E Malavolta M . Glymphatic system and psychiatric disorders: need for a new paradigm? Front Psychiatry. (2025) 16:1642605. doi: 10.3389/fpsyt.2025.1642605, PMID: 41425825 PMC12711785

[5] Wang ZW Ma XC Xiao CL . Standardized treatment strategy for depressive disorder. Adv Exp Med Biol. (2019) 1180:193–99. doi: 10.1007/978-981-32-9271-0_10, PMID: 31784964

[6] Rost K Nutting P Smith JL Elliott CE Dickinson M . Managing depression as a chronic disease: a randomised trial of ongoing treatment in primary care. BMJ. (2002) 325:934. doi: 10.1136/bmj.325.7370.934, PMID: 12399343 PMC130058

[7] Rush AJ Trivedi M Carmody TJ Biggs MM Shores-Wilson K Ibrahim H . One-year clinical outcomes of depressed public sector outpatients: a benchmark for subsequent studies. Biol Psychiatry. (2004) 56:46–53. doi: 10.1016/j.biopsych.2004.04.005, PMID: 15219472

[8] Akil H Gordon J Hen R Jacitch J Mayberg H McEwen B . Treatment resistant depression:a multi-scale, systems biology approach. Neurosci Biobehav Rev. (2018) 84:272–88. doi: 10.1016/j.neubiorev.2017.08.019, PMID: 28859997 PMC5729118

[9] Gobbi G Ghabrash MF Nuñez N Tabaka J Sante JD Saint-Laurent M . Antidepressant combination versus antidepressants plus second-generation anti-psychotic augmentation in treatment-resistant unipolar depression. Int Clin Psychopharmacol. (2018) 33:34–43. doi: 10.1097/YIC.0000000000000196, PMID: 28906325

[10] Kudlow PA McIntyre RS Lam RW . Early Switching Strategies in Antidepressant Non-Responders: current evidence and future research directions. CNS Drugs. (2014) 28:601–09. doi: 10.1007/s40263-014-0171-5, PMID: 24831418

[11] Ogle NR Akkerman SR . Guidance for the discontinuation or switching of antidepressant therapies in adults. J Pharm Pract. (2013) 26:389–96. doi: 10.1177/0897190012467210, PMID: 23459282

[12] Bschor T Kern H Henssler J Baethge C . Switching the Antidepressant after non-response in adults with major depression:a systematic literature search and meta-analysis. J Clin Psychiatry. (2018) 79:16r10749. doi: 10.4088/JCP.16r10749, PMID: 27929611

[13] Hofmann P . Watchful waiting when treating depression:Still our maxim? J fur Neurologie Neurochirurgie und Psychiatr. (2014) 15:220–23.

[14] Feng Y Xiao L Wang WW Ungvari GS Ng CH Wang G . Guidelines for the diagnosis and treatment of depressive disorders in China:the second edition. J Affect Disorder. (2019) 253:352–56. doi: 10.1016/j.jad.2019.04.104, PMID: 31078835

[15] Malhi GS Bell E Bassett D Boyce P Bryant R Hazell P . The 2020 Royal Australian and New Zealand College of Psychiatrists clinical practice guidelines for mood disorders. Aust NZJ Psychiatry. (2021) 55:7–117. doi: 10.1177/0004867420979353, PMID: 33353391

[16] Kendrick T Pilling S Mavranezouli I Megnin-Viggars O Ruane C Eadon H . Management of depression in adults:summary of updated NICE guidance. BMJ. (2022) 378:01557. doi: 10.1136/bmj.o1557, PMID: 35858703

[17] Pannu A Goyal RK . The potential role of dopamine pathways in the pathophysiology of depression: current advances and future aspects. CNS Neurol Disord Drug Targets. (2025) 24:340–52. doi: 10.2174/0118715273357909241126064951, PMID: 39639477

[18] Rizvi SJ Grima E Tan M Rotzinger S Lin P Mcintyre RS . Treatment-resisitant depression in primary care a across Canada. Can J Psychiatry. (2014) 56:349–57. doi: 10.1136/bmj.o1557, PMID: 25007419 PMC4086317

[19] Martinotti G Vita A Fagiolini A Maina G Bertolino A Dell’Osso B . Real-world experience of esketamine use to manage treatment-resistant depression: A multicentric study on safety and effectiveness (REAL-ESK study). J Affect Disord. (2022) 319:646–54. doi: 10.1016/j.jad.2022.09.043, PMID: 36167246

[20] Pettorruso M Guidotti R d’Andrea G De Risio L D’Andrea A Chiappin S . Predicting outcome with Intranasal Esketamine treatment: A machine-learning, three-month study in Treatment-Resistant Depression (ESK-LEARNING). Psychiatry Res. (2023) 327:115378. doi: 10.1016/j.psychres.2023.115378, PMID: 37574600

[21] Di Nicola M Pepe M d`Andrea G Marcelli I Pettorruso M Andriola I . Patient experience with intranasal esketamine in treatment-resistant depression: insights from a multicentric Italian study (REAL-ESKperience). J Pers Med. (2025) 15:161. doi: 10.3390/jpm15040161, PMID: 40278340 PMC12029048

[22] Subbaiah MAM . Triple reuptake inhibitors as potential therapeutics for depression and other disorders: design paradigm and developmental challenges. J Med Chem. (2018) 61:2133–65. doi: 10.1021/acs.jmedchem.6b01827, PMID: 28731336

[23] Zhu HB Wang WY Sha CJ Guo W Li CM Zhao FJ . Pharmacological Characterization of toludesvenlafaxine as a triple reuptake Inhibitor. Front Pharmacol. (2021) 12:741794. doi: 10.3389/fphar.2021.741794, PMID: 34594228 PMC8476831

[24] Zhou SZ Li P Lyu XZ Lai XF Liu ZX Zhou JW . Efficacy and dose-response relationships of antidepressants in the acute treatment of major depressive disorders: a systematic review and network meta-analysis. Chin Med J (Engl). (2025) 138:1433–38. doi: 10.1097/CM9.0000000000003138, PMID: 38902199 PMC12180811

[25] Mi WF Di XL Wang YM Li HF Xu XF Li LH . A phase 3, multicenter, double-blind, randomized, placebo-controlled clinical trial to verify the efficacy and safety of toludesvenlafaxine (LY03005) for major depressive disorder. Transl Psychiatry. (2023) 13:163. doi: 10.1038/s41398-023-02435-0, PMID: 37164957 PMC10171157

[26] Malhi GS Hitching R Berk M Boyce P Porter R Fritz K . Pharmacological management of unipolar depression. Acta Psychiatr Scand Suppl. (2013) 443:6–23. doi: 10.1111/acps.12122, PMID: 23586873

[27] Montgomery SA Asberg M . A new depression scale designed to be sensitive to change. Br J Psychiatry. (1979) 134:382–89. doi: 10.1192/bjp.134.4.382, PMID: 444788

[28] Hamilton M . The assessment of anxiety states by rating. Br J Med Psychol. (1959) 32:50–5. doi: 10.1111/j.2044-8341.1959.tb00467.x, PMID: 13638508

[29] Rizvi SJ Quilty LC Sproule BA Cyriac A Bagby RM Kennedy SH . Development and validation of the Dimensional Anhedonia Rating Scale (DARS) in a community sample and individuals with major depression. Psychiatry Res. (2015) 229:109–19. doi: 10.1016/j.psychres.2015.07.062, PMID: 26250147

[30] Endicott J Nee J Harrison W Blumenthal R . Quality of Life Enjoyment and Satisfaction Questionnaire:a new measure. Psychopharmacol Bull. (1993) 29:321–6., PMID: 8290681

[31] Kadouri A Corruble E Falissard B . The improved Clinical Global Impression Scale (iCGI): development and validation in depression. BMC Psychiatry. (2007) 6:7. doi: 10.1186/1471-244X-7-7, PMID: 17284321 PMC1802073

[32] MeGahuey CA Gelenberg AJ Laukes CA Moreno FA Delgado PL Mcknight KM . The Arizona sexual experience scale (ASEX): reliability and validity. Sex Marital Ther. (2000) 26:25–40. doi: 10.1080/009262300278623, PMID: 10693114

[33] Cipriani A Furukawa TA Salanti G Chaimani A Atkinson LZ Ogawa Y . Comparative efficacy and acceptability of 21 antidepressant drugs for the acute treatment of adults with major depressive disorder: a systematic review and network meta-analysis. Lancet. (2018) 391:1357–66. doi: 10.1016/S0140-6736(17)32802-7, PMID: 29477251 PMC5889788

[34] Celada P Bortolozzi A Artigas F . Serotonin 5-HT1A receptors as targets for agents to treat psychiatric disorders:rationale and current status of research. CNS Drugs. (2013) 27:703 –16. doi: 10.1007/s40263-013-0071-0, PMID: 23757185

[35] Tunvirachaisakul C Gould RL Coulson MC Ward EV Reynolds G Gathercole RL . Predictors of treatment outcome in depression in later life:A systematic review and meta-analysis. J Affect Disord. (2018) 227:164–82. doi: 10.1016/j.jad.2017.10.008, PMID: 29100149

[36] Vinckier F Gourion D Mouchabac S . Anhedonia predicts poor psychosocial functioning:Results from a large cohort of patients treated for major depressive disorder by general practitioners. Eur Psychiatry. (2017) 44:1–8. doi: 10.1016/j.eurpsy.2017.02.485, PMID: 28535406

[37] Gaynes BN Rush AJ Trivedi MH Wisniewski SR Spencer D Fava M . The STAR*D study: treating depression in the real world. Cleve Clin J Med. (2008) 75:57–66. doi: 10.3949/ccjm.75.1.57, PMID: 18236731

[38] Warden D Rush AJ Trivedi MH Fava M Wisniewski SR . The STAR*D Project results: a comprehensive review of findings. Curr Psychiatry Rep. (2007) 9:449–59. doi: 10.1007/s11920-007-0061-3, PMID: 18221624

[39] Iosifescu DV Jones A O’Gorman C Streicher C Feliz S Fava M . Efficacy and safety of AXS-05 (Dextromethorphan-bupropion) in patients with major depressive disorder: A phase 3 randomized clinical trial (GEMINI). J Clin Psychiatry. (2022) 83:21m14345. doi: 10.4088/JCP.21m14345, PMID: 35649167

[40] Arnaud A Benner J Suthoff E Werneburg B Reinhart M Sussman M . The impact of early remission on disease trajectory and patient outcomes in major depression disorder (MDD): A targeted literature review and microsimulation modeling approach based on the Sequenced Treatment Alternatives to Relieve Depression (STAR*D) study. J Affect Disord. (2023) 325:264–72. doi: 10.1016/j.jad.2022.12.147, PMID: 36608852

[41] Huang ZW Wu JH Guan YH Wei YM Xie F Shen YF . PET/CT study of dopamine transporter (DAT) binding with the triple reuptake inhibitor toludesvenlafaxine in rats and humans. Eur J Nucl Med Mol Imaging. (2024) 51:2638–48. doi: 10.1007/s00259-024-06700-2, PMID: 38587645

[42] Dormegny-Jeanjean LC Billy C Mainberger O Weibel S Schorr B Obrecht A . Potential efficacy of dopaminergic antidepressants in treatment resistant anergic-anhedonic depression results of the chronic anergic-anhedonic depression open trial-CADOT. Front Psychiatry. (2023) 14:1194090. doi: 10.3389/fpsyt.2023.1194090, PMID: 37829759 PMC10565009

[43] Guiard BP Chenu F Mansari ME Blier P . Characterization of the electrophysiological properties of triple reuptake inhibitors on monoaminergic neurons. Int J Neuropsychopharmacol. (2011) 14:211–23. doi: 10.1017/S1461145710000076, PMID: 20149268

[44] Britta H Heinz B . Depression and antidepressants:insights from knockout of dopamine, serotonin or noradrenaline re-uptake transporters. Pharmacol Ther. (2011) 129:352–68. doi: 10.1016/j.pharmthera.2010.12.002, PMID: 21147164

[45] Kirsch I Deacon BJ Huedo-Medina TB Scoboria A Moore TJ Johnson BJ . Initial severity and antidepressant benefits: a meta-analysis of data submitted to the Food and Drug Administration. PLoS Med. (2008) 5:e45. doi: 10.1371/journal.pmed.0050045, PMID: 18303940 PMC2253608

[46] Sampogna G Toni C Catapano P Rocca BD Vincenzo MD Luciano M . New trends in personalized treatment of depression. Curr Opin Psychiatry. (2024) 37:3–8. doi: 10.1097/YCO.0000000000000903, PMID: 37865845

